# Goals and Habits in the Brain

**DOI:** 10.1016/j.neuron.2013.09.007

**Published:** 2013-10-16

**Authors:** Ray J. Dolan, Peter Dayan

**Affiliations:** 1 Wellcome Trust Centre for Neuroimaging, Institute of Neurology, University College London, London WC1 3BG, UK; 2 Gatsby Computational Neuroscience Unit, University College London, London WC1N 3AR, UK

## Abstract

An enduring and richly elaborated dichotomy in cognitive neuroscience is that of reflective versus reflexive decision making and choice. Other literatures refer to the two ends of what is likely to be a spectrum with terms such as goal-directed versus habitual, model-based versus model-free or prospective versus retrospective. One of the most rigorous traditions of experimental work in the field started with studies in rodents and graduated via human versions and enrichments of those experiments to a current state in which new paradigms are probing and challenging the very heart of the distinction. We review four generations of work in this tradition and provide pointers to the forefront of the field’s fifth generation.

## Teaser

Dolan and Dayan provide a computational perspective on two fundamental forms of control, goal directed and habitual. They discuss how these forms of control are expressed in the human brain and how dominance of one or other of these systems may contribute to psychopathology.

## Main Text

### Introduction

An important and pervasive idea in the psychology of decision making and choice is that there is more than one class of possible strategy for acting. A key division is between forms of reflective control, which depend on the more or less explicit consideration of possible prospective future courses of actions and consequent outcomes, and forms of reflexive control a term we use in the restricted sense to describe how retrospective experience with good and bad outcomes sculpts present choice.

This apparent dichotomy is so intuitively obvious that it has been realized in many, slightly different, and only partly compatible, ways (Dickinson, 1985; Kahneman, 2011; Stanovich and West, 2002). Here, we single out one particular strand that has arguably been the most fecund in cognitive and theoretical neuroscience, providing a set of behaviorally rigorous criteria for separating out the two classes of control. In turn, this has led to a set of important studies into the partly distinct neural realizations of these classes and thence to an understanding of their computational and statistical characteristics. The latter provides a normative rationale for their coexistence as offering efficient solutions to the demands of complex and changing environments and has also underpinned the design and interpretation of a collection of targeted empirical studies.

We review the evolution of this strand by considering five generations of studies. We use the term “generation” as a frame of reference for our discussion and apply a liberal semantic license in our use of the term, using it to describe a sequential evolution of ideas, as opposed to an orderly sequence in epochs of time. The zeroth generation represents some of the earliest intellectual battles in psychology between advocates of cognitive maps and stimulus-response theories (Thorndike, 1911; Tolman, 1948). The fallout from this debate was a first generation of behaviorally rigorous studies of goal-directed and habitual instrumental control, which in turn provided the foundation for investigation of their neural realizations (Balleine and Dickinson, 1998; Balleine, 2005; Dickinson and Balleine, 2002; Killcross and Coutureau, 2003). In the second generation, these paradigms were carefully adapted for human neuroimaging studies, validating and amplifying the results from rodents (Tanaka et al., 2008; Liljeholm et al., 2011; Tricomi et al., 2009; Valentin et al., 2007). In the third and fourth generations, an analysis of the two forms of control in terms of model-based and model-free reinforcement learning (Doya, 1999; Doya et al., 2002; Sutton and Barto, 1998; Daw et al., 2005) was used to realize new tasks and to provide powerful methods for interpreting the ensuing results. The third generation crystallized the differences in a computationally transparent manner; the fourth generation made further changes to provide insight into the ongoing cooperation and competition between these systems (Fermin et al., 2010; Doll et al., 2009; Gershman et al., 2012; Daw et al., 2011; Gläscher et al., 2010; Otto et al., 2013; Simon and Daw, 2011; Wunderlich et al., 2012a, 2012b). Finally, we highlight the immediate horizon of questions that we surmise are now being, or perhaps are about to be, addressed by a fifth generation of investigations. Note that new work also continues in generations one to four, with the youthful exuberance of the later ones complementing the sage wisdom of the earlier.

In this Review, we primarily focus on human instrumental behavior. There are excellent reviews of habitual and goal-directed behavior that cover an extensive animal literature (Balleine, 2005; Dickinson and Balleine, 1994; Dickinson and Charnock, 1985). Consequently, these animal studies are only sketched in so far as they provide an essential background to our Review of the relevant human data. Many of the issues that we lack space to discuss are treated by others (Rangel et al., 2008; Botvinick, 2012; Berridge, 2001; Padoa-Schioppa and Assad, 2006; Daw et al., 2006a; Dayan and Daw, 2008; Balleine and O’Doherty, 2010; Yin and Knowlton, 2006; Maia, 2009; Niv, 2009; Doll et al., 2012).

### Generation 0: Cognitive Maps

In a famous paper, the psychologist Edward Tolman considered a typical learning experiment involving rats negotiating a maze environment to reach a rewarded goal state (Tolman, 1948). This was a time of substantial theoretical debate, and though all could agree on the basic facts that with increasing experience, animals made fewer and fewer errors in reaching the goal state and took less and less time to do so, there were nevertheless starkly polarized views on the underlying cause.

Stimulus-response (S-R) theories, the bedrock of psychology in the first half of the 20^th^ century, insisted that instrumental behavior reflected the emergence of an associative structure, wherein representations of a stimulus context during learning became, with increasing experience, more strongly connected to a mechanism generating behavioral responses. A favored analogy was that of a complicated telephone switchboard acting so as to couple incoming sensory signals to outgoing effectors. This seductive narrative reduced to the idea, as caricatured by Tolman, that learning resulted in an animal coming to respond more and more “helplessly” to a succession of external and internal stimuli that “call out the walkings, runnings, turnings, retracing, smellings, rearings and the like which appear” (Tolman, 1948). Tolman argued strongly against what he considered the fundamental poverty in this type of account. Rather, he aligned himself with so-called “field theorists” (Tolman, 1948), who proposed that animals learn such a maze task by forming “a field map of the environment,” more commonly referred to these days as a cognitive map (O’Keefe and Nadel, 1978), which then provides the necessary guidance mechanism for the observed learning.

This dispute led to the design of critical experiments, for instance, examining the nature of learning that occurs in the absence of the driving force of reinforcement. The classic example here was the observation that an animal left to explore a maze environment, without ever experiencing a reinforcing reward contingency, can nevertheless be shown to be engaging in what is known as latent learning (Blodgett, 1929; Thistlethwaite, 1951). Latent learning is “unmasked” when the animal is subsequently tasked to navigate toward a rewarded goal state in this same environment. Critically, pre-exposed animals show facilitation in learning relative to naive animals, suggesting that the preceding nonrewarded exposure epochs foster the formation of a cognitive map that aids subsequent attainment of the rewarded goal location (Tolman and Honzik, 1930). Latent learning about outcomes is also observed in procedures such as the irrelevant incentive effect (Krieckhaus and Wolf, 1968). Consider two groups of animals trained when thirsty, but not salt deprived, to press a lever to get either water or a sodium solution. Members of the latter group are found to press more avidly than those of the former when subsequently salt deprived, even if lever pressing is in extinction (when no solution of either sort is provided). This shows that latent learning occurred in relation to the salt characteristics of the solution, even when it was irrelevant in the context of the then prevailing motivational state.

At the time of the early studies, it was not easy to quantify how complicated the latent learning tasks were that the animals were being asked to perform. These experiments substantially predated the invention of dynamic programming (Bellman, 1957), which helped formalize the whole domain. The resulting theory, and particularly a computational variant called reinforcement learning (Sutton and Barto, 1998), has underpinned much of the impact of computational modeling in the later generations of studies that has resulted in a considerable sharpening of experimental design and analysis.

In terms of behavioral control, a cognitive map can be seen as a representational template that enables an animal, through mental search, to find the best possible action at a particular state. Some indirect evidence about search came from what is termed “vicarious trial and error” (VTE), a class of behavior evident at choice points that is manifest as motor hesitations and repetitive looking back and forth (Muenzinger, 1938). VTE behaviors are not merely incidental, since animals that express more VTE behaviors turn out to be better learners (Muenzinger, 1938; Tolman, 1938). Furthermore, a diminution in the frequency of VTE over the course of learning was taken as evidence that learning involved a shift away from reliance on a form of search through a cognitive map toward more automatic forms of control (Muenzinger, 1938; Tolman, 1938).

The idea of a cognitive map, evidently a revolutionary notion in the early part of the last century, is now key to much theorizing in cognitive neuroscience. Cognitive maps occupy a central role in contemporary ideas related to active memory or prospection (Schacter et al., 2007), where the hippocampus (O’Keefe and Nadel, 1978) has been shown to play a critical role (O’Keefe and Nadel, 1978). For instance, human subjects with hippocampal lesions, when tasked to imagine possible future states, manifest a profound impairment in self-projection or prospection (Hassabis et al., 2007). Equally, in rats the expression of VTE behaviors is abolished by hippocampal lesions (Hu and Amsel, 1995). Furthermore, one of the most famous findings about the hippocampus in rats is the existence of place cells, which provide a population code for representing space (O’Keefe and Nadel, 1978). These cells are known to be activated at choice points in a way consistent with internal exploration of future possibilities, possibly coupled to VTEs (Johnson and Redish, 2007; Pfeiffer and Foster, 2013; van der Meer and Redish, 2009). Note, though, as we discuss below, structures other than the hippocampus are also implicated; these include distinct prefrontal cortical regions and possibly the basolateral nucleus of the amygdala and dorsomedial striatum (Balleine and Dickinson, 1998; Corbit and Balleine, 2003; Yin et al., 2005; Balleine, 2005).

### Generation 1: Goal-Directed Actions and Habits

These early studies established an attractive dichotomy between control based on a cognitive map and control based on S-R associations. With the decrease in VTE behavior as a function of experience, they even offered the prospect of a transition from map-based to S-R-based determination, consistent with the long-standing observation that repetition endows a high degree of motoric fluency to even the most complex action sequences (James, 1890; Kimble and Perlmuter, 1970). However, short of using virtual reality, it is hard to achieve stimulus control in navigational domains, and it remains possible that spatial behavior may depend on special-purpose mechanisms of geometrical cognition (Gallistel, 1990; Burgess, 2008; Cheng, 1986; O’Keefe and Nadel, 1978) or indeed Pavlovian approach, for which the contingency between action and outcome is moot (Mackintosh, 1983). Therefore, the first generation of analytical studies operationalized the use of a cognitive map in a nonspatial domain as goal-directed behavior, which it then contrasted with the notion of a habit (Dickinson and Balleine, 1994, 2002; Balleine and Dickinson, 1998; Graybiel, 2008; Adams and Dickinson, 1981; Dickinson and Charnock, 1985).

Instrumental behavior is considered goal directed if it meets two criteria. First, it should reflect knowledge of the relationship between an action (or sequence of actions) and its consequences. This is known as response-outcome or R-O control. Second, the outcome should be motivationally relevant or desirable at the moment of choice. Crudely, subjects choose actions because they think that those actions lead to outcomes that they presently desire. By contrast, habitual instrumental behavior is supposed to have been stamped in by past reinforcement (Thorndike, 1911) and so is divorced from the current value of an associated outcome. Thus, key characteristics of habitual instrumental control include automaticity, computational efficiency, and inflexibility, while characteristics of goal-directed control include active deliberation, high computational cost, and an adaptive flexibility to changing environmental contingencies (Dayan, 2009).

Demonstrating that behavior is goal directed is usually assayed in a test session using posttraining manipulations, which either involve reinforcer devaluation or contingency degradation. Consider a test session carried out in extinction, i.e., without ongoing reinforcement. In this case, there should be less instrumental responding for an outcome that has been devalued (for example, a food reinforcer that has just been rendered unpalatable) than for an outcome that has not. Importantly, this is only true if knowledge of a reinforcer’s current value (i.e., its desirability) exerts a controlling influence on performance; in other words, if task performance is mediated by a representation of the reinforcer (Adams and Dickinson, 1981). Conversely, habitual behavior comprises instrumental responding that continues to be enacted even when the outcome is undesired. Various circumstances promote habitual responding, notably extended training on interval schedules of reinforcement involving single actions and single outcomes (Dickinson and Charnock, 1985; Dickinson and Balleine, 2002; Dickinson et al., 1983). The requirement for extensive experience is key and this also implies that behavior is initially goal directed but then becomes habitual over the course of experience. For completeness, we also mention the contingency criterion wherein goal-directed behavior also involves an encoding of the causal relationship between actions and their consequences. Consider a subject trained to press a lever to receive an outcome. If the outcome subsequently becomes equally available with and without a lever press, goal-directed control leads to a decrease in pressing (Dickinson and Balleine, 1994; Dickinson and Charnock, 1985).

The behavioral distinction between goal-directed and habitual control has provided the foundation for a wealth of lesion, inactivation, and pharmacological animal experiments investigating their neural bases. Rodent studies repeatedly highlight a dorsomedial striatum circuit that supports goal-directed behavior (Balleine, 2005; Corbit and Balleine, 2005; Yin et al., 2005). Related studies show that a circuit centered on dorsolateral striatum supports habit-based behavior (Yin et al., 2004, 2005; White, 1997; Balleine, 2005; Balleine and Dickinson, 1998; Killcross and Coutureau, 2003; Yin and Knowlton, 2006). Lesions to dorsolateral striatum result in a maintenance of goal-directed behavior even with extended training, a pattern that contrasts with the effect of lesions to dorsomedial striatum that result in an early emergence of habitual behavior (Yin and Knowlton, 2006). There is also explicit evidence for the transfer from dorsomedial to dorsolateral over the course of training (Belin et al., 2009; Graybiel, 2008; Yin et al., 2009; Thorn et al., 2010).

Behavioral dissociations that mirror precisely those seen following striatal lesions are also seen with lesions to distinct sectors of prefrontal cortex, a testament to the close functional affinity of these regions. Frontal prelimbic lesions abolish sensitivity to both outcome devaluation manipulations as well as to degradation of instrumental contingency (Balleine and Dickinson, 1998; Corbit and Balleine, 2003). Pretraining, but not posttraining, lesions disrupt acquisition, but not expression, of goal-directed behavior (Ostlund and Balleine, 2005). Likewise, a reversible inactivation targeting infralimbic medial prefrontal cortex impacts on the expression of habitual behavior (Coutureau and Killcross, 2003). Furthermore, selective lesions to prelimbic medial prefrontal cortex induce lack of sensitivity to goal value following either limited or extended training, whereas selective lesions to infralimbic regions result in an opposite deficit, namely retained sensitivity to goal value after both limited and extended training (Killcross and Coutureau, 2003).

The fact that prelimbic prefrontal cortex and dorsomedial striatum both support goal-directed action is in line with the anatomical connectivity between these regions (Groenewegen et al., 1990; McGeorge and Faull, 1989). The connection between infralimbic cotex and dorsolateral striatum is rather less clear and in the rat, caudal, but not rostral, infralimbic cortex projects to ventral parts of medial caudate putamen (Vertes, 2004), but there is no known projection to dorsolateral striatum. Thus, one possible locus for interaction is through indirect connections via the ventral striatum, the amygdala, the substantia nigra, or by way of projections to other cortical areas and thence to dorsolateral striatum (Hurley et al., 1991). It is known that the activity of ensembles of neurons in dorsolateral striatum and the infralimbic cortex reflect the creation and stabilization of habits, with interesting differences between the regions in the evolution of these patterns (Smith and Graybiel, 2013). However, it needs to be acknowledged that there is, as yet, no consensus as to what constitutes the homologous area in primates to rat infralimbic cortex.

This double dissociation makes a strong case that prelimbic regions are crucial for goal-directed performance, while infralimbic lesions prevent the emergence of habitual responding that overrides an initial dominance in goal-directed responding. However, it is likely that in the intact animal, there is a dynamic interdependency between goal-directed and habitual systems and that control is likely to emerge simultaneously and competitively (Wassum et al., 2009). If habit and goal-directed processes indeed act concurrently, then this invites questions regarding what precisely are the factors that influence the integration and competition between the two systems. We return to these issues below. It is also worth noting here that although goal-directed or response-outcome learning has a strong declarative flavor, it is conceptually distinct from a hippocampal-dependent stimulus-stimulus form of learning.

There are some alluring parallels with this account of the evolution from goal-directed to habitual responding. One is the transfer of control of a simple spatial behavior (turning in a “plus” maze) from a hippocampal-dependent, allocentric, reference frame to a striatum-dependent, egocentric one (Packard and McGaugh, 1996). Similar double dissociations arise from reversible lesions in these two regions at different time points, for example early or late, during learning. The other parallel is with the transfer over the course of experience from allocentric to egocentric reference frames of a sequence of manual button presses (Hikosaka et al., 1999), although this was proposed to depend on two separate cortical regions that both interact with the basal ganglia. Recent lesion studies have examined more sophisticated representational issues, for instance, comparing the sort of stimulus-response associations that underpin habits to a hierarchical association scheme in which the presence of a certain stimulus implies that a response leads to a particular outcome (Bradfield and Balleine, 2013). Control apparently based on the latter representation is compromised by lesions to posterior dorsomedial striatum, whereas in complex circumstances, lesions to dorsolateral striatal actually enhanced learning, suggesting that a form of competition might be at work.

### Generation 2: Actions and Habits in the Human Brain

The rich backdrop of animal experiments has inspired a collection of studies that address the architecture of human instrumental control, often employing straightforward adaptations of successful animal paradigms as well as seeking and exploiting homologies (Balleine and O’Doherty, 2010; Haber and Knutson, 2010). Many of these have involved the use of fMRI in order to investigate the neural representation of the value of stimuli and actions to see whether or not they are affected by devaluation.

We consider two studies of particular interest in this context that respectively target goal-directed and habitual choice (Valentin et al., 2007; Tricomi et al., 2009). Valentin and colleagues trained human subjects on a task in which two different instrumental actions resulted in two distinct food reward outcomes (Valentin et al., 2007). One of the outcomes was then devalued (by feeding subjects that food to satiety, i.e., until they would consume no more of it). As expected from the moderate amount of initial training, behavior was goal directed, with actions leading to the devalued outcome being selectively depressed in extinction. Of note was the observation that the BOLD signal in a ventral sector of orbitofrontal cortex decreased for a devalued compared to a nondevalued action, leading the authors to conclude that this region plays a role in goal-directed choice. Indeed, there has been much work in humans, nonhuman primates, and rodents suggesting that this region plays a key role in representing the sort of values that underpin goal-directed control (Daw et al., 2006b; Gottfried and Dolan, 2004; Hampton et al., 2006; Padoa-Schioppa and Assad, 2006; Schoenbaum and Roesch, 2005; Thorpe et al., 1983). vmPFC is likely to have a complex role in value representation and there is strong evidence linking this region to both stimulus value and outcome value, and even recent evidence linking it to action value (FitzGerald et al., 2012). We note also that human lesion data has led to the suggestion that orbital prefrontal cortex implements encoding of stimulus value with dorsal cingulate cortex implementing encoding of action value (Camille et al., 2011).

Tricomi and colleagues set out to investigate the emergence of habitual behavior (Tricomi et al., 2009). Subjects were trained on action-outcome reward contingencies that mirrored a free-operant paradigm in the animal literature, where one group of subjects had extensive training, and another had little training. After outcome devaluation, performance showed that the minimally trained group retained outcome sensitivity, while the extensively trained group did not, just as in the animal studies. A within-group analysis of fMRI data from the extensively trained subjects comparing later sessions (when behavior was habitual) to earlier sessions (when it would likely have been goal directed) highlighted increased cue-related activity in right posterior putamen/globus pallidum, consistent with the rodent findings showing involvement of the dorsolateral striatum in habitual responding.

### Generation 3: Model-Based and Model-free Analyses

Along with these experimental results, the conceptual precision of goal-directed and habitual decision making invited the ascription of computational accounts to both of them and to their potential interactions. These models in turn led to the design of novel experimental paradigms that cast new light on the dichotomy.

The basis of the models is the normative account of instrumental control that comes from the field of reinforcement learning (RL). This is based on dynamic programming (Bellman, 1957) and brings together ideas from artificial intelligence, optimal control theory, operations research, and statistics to understand how systems of any sort can learn to choose actions that maximize reward and minimize punishments (Sutton and Barto, 1998).

Typical RL problems involve four key quantities: (1) states, which can be thought of as contexts or stimuli; (2) actions that are available at or given by these states; (3) transitions between states that are occasioned (perhaps stochastically) by actions; and (4) utilities, which quantify the immediate worth of states in terms of reward or punishments. The utilities depend on the motivations of the subject (water is more valuable given thirst). The subject has to find a good policy—i.e., a good choice of action at each state—that optimizes the long-run worth of all the utilities that will be collected. All the tasks discussed above can be mapped onto this framework in a straightforward manner.

Two ends of a spectrum of RL methods are model-based and model-free control (where the term model refers to a mental as opposed to a computational model); it is these that have been associated with goal-directed and habitual control, respectively (Daw et al., 2005; Doya et al., 2002). As we noted, goal-directed control is based on working out, and then evaluating, the outcomes associated with a long-run sequence of actions. Model-based control conceives of this in terms of sophisticated, computationally demanding, prospective planning, in which a decision tree of possible future states and actions is built using a learned internal model of the environment. The current state is the root, and the policy with the highest value is determined by searching the tree either forward from the root to the leaves (the terminal points) or backward from the leaves to the root, accumulating utilities along the way to quantify the long-run worth. This search process can be thought of as an expression of a form of mental simulation (Chersi and Pezzulo, 2012; Doya, 1999; Hassabis et al., 2007; Johnson and Redish, 2007; Pfeiffer and Foster, 2013; Schacter et al., 2012). Critically, the idea that prospective outcomes are explicitly represented allows these states to be valued (putatively via the orbitofrontal or ventromedial prefrontal cortex) (Valentin et al., 2007; Fellows, 2011; O’Doherty, 2011) according to their current worth and so choices can be immediately sensitive to devaluation. Equally, given information that the transitions have changed, as in contingency degradation, the decision tree and the associated optimal choices will adapt straightaway. The tree is just like a cognitive map, one that enables the flexible consideration of the future consequence of actions (Thistlethwaite, 1951). It is easy to appreciate that building and evaluating a tree imposes processing and working memory demands that rapidly become unrealistic with increasing depth. Consequently, a model-based agent is confronted with overwhelming computational constraints that in psychological terms reflect the known capacity limitations within attention and working memory.

By contrast, model-free control involves a particular sort of prediction error, the best known example of which is the temporal difference (TD) prediction error (Sutton, 1988). Predictions at one step are supposed to be of the long-run sequence of actions or states starting at that step, and so the ideal prediction error would measure the difference between the amount of utility that is actually delivered over that long run and the amount that is predicted. However, waiting to experience all those utilities in the long run is usually impossible. The TD prediction error obviates this requirement via the trick of using the prediction at the next step to substitute for the remaining utilities that are expected to arrive and it is this aspect that leads it to sometimes be seen as forward looking. In total, this prediction error is based on the utilities that are actually observed during learning and trains predictions of the long-run worth of states, criticizing the choices of actions at those states accordingly. Further, the predictions are sometimes described as being cached, because they store experience. Much evidence points to phasic activity of dopamine neurons as reporting an appetitive prediction error (Schultz et al., 1997; Montague et al., 1996).

**Figure 1:**
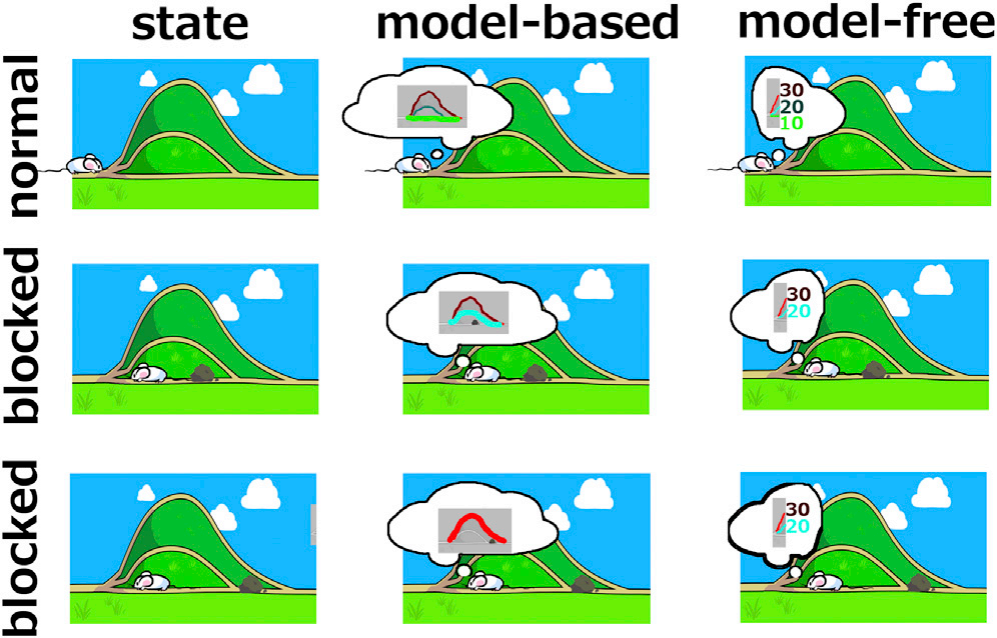
Schematic of the Tolman Detour Task Model-based and model-free decision making in a cartoon of a maze invented by Tolman and Honzik (1930). Left column: the maze has three paths (long, medium, and short), but a boulder can block just the short path (middle; after the subject has found the boulder and comes back to the start) or both short and medium (bottom). Middle column: the model-based system uses a model (thought bubble) of the maze to plan; after discovering the boulder, it knows whether the medium path is open (middle; cyan is best) or blocked (bottom; red is best). Right column: the model-free system learns path lengths based on extensive experience; if no path is blocked, this leads to the optimal choice (top; green is best); when it discovers the boulder by going along the short, green, path, it only knows that this path is blocked and thus tries the medium path (cyan) whether it is viable (middle) or not (bottom) (figure design by Alyssa Dayan).

Model-free control is computationally efficient, since it replaces computation (i.e., the burdensome simulation of future states) with memory (i.e., stored discounted values of expected future reward); however, the forward-looking nature of the prediction error makes it statistically inefficient (Daw et al., 2005). Further, the cached values depend on past utilities and so are divorced from the outcomes that they predict. Thus, model-free control is fundamentally retrospective, and new cached values, as might arise with a change in the utility of an outcome in an environment, can only be acquired through direct experience. Thus, in extinction, model-free control, like habitual control, has no immediate sensitivity to devaluation (Figure 1).

Initial human imaging studies that used RL methods to examine the representation of values and prediction errors largely focused on model-free prediction and control, without worrying about model-based effects (Berns et al., 2001; O’Doherty, 2004; O’Doherty et al., 2003; Haruno et al., 2004). These showed that the BOLD signal in regions of dorsal and ventral striatum correlated with a model-free temporal difference prediction error, the exact type of signal thought to be at the heart of reinforcement learning. A huge wealth of subsequent studies have confirmed and elaborated this picture.

More recently, a plethora of paradigms has provided as sharp a contrast between model-free and model-based for human studies as animal paradigms have between goal-directed and habitual control. One set of examples (Daw et al., 2011; Gläscher et al., 2010) is based on a sequential two-choice Markov decision task, in which the action at the first state is associated with one likely and one unlikely transition. Model-free control simply prefers to repeat actions that lead to reward, irrespective of the likelihood of that first transition. By contrast, model-based control, because it builds the decision tree, can correctly ascribe those rewards following a rare transition to an alternative (nonselected) action—which, despite not predicting reward on the current trial, will be more likely to lead to reward on future trials. This key difference makes it possible to discern the influence of each controller on behavior and also to determine whether neural signals are correlated with predictions and prediction errors specific to each controller.

Motivated by Tolman and Honzik (Tolman and Honzik, 1930), Gläscher and colleagues employed a variant of this task to examine latent learning (Gläscher et al., 2010). Subjects were extensively taught the first-state transitions and were then told the utilities at the second state. Appropriate initial behavior in the task once the utilities were revealed could only arise from model-based control. However, the authors observed that the initial supremacy of model-based controller declined rather precipitately over time, even in the absence of information that would contradict this controller (Gläscher et al., 2010). This decline was suggested as an analog of fast acquisition of habitual behavior. During the interregnum, behavior was best fit by a hybrid model in which both systems exerted some control. fMRI data highlighted a conventional model-free temporal difference reward prediction error in ventral striatum, whereas a different sort of state prediction error, associated with the acquisition of the model, was seen in posterior inferior parietal and lateral prefrontal cortices.

Daw and colleagues devised a different variant of the task to encourage a stable balance between model-based and model-free control (Daw et al., 2011). The logic of the task was that model-based and model-free strategies for RL predict different patterns by which reward obtained in the second stage should impact first-stage choices on subsequent trials. Consider a trial in which a first-stage choice, uncharacteristically, led to a second stage state with which it is not usually associated, and the choice then made at the second stage turned out to be rewarded. Model-free reinforcement predicts that this experience will increase the probability of repeating the successful first-stage choice. By contrast, if a subject chooses using an internal model of the transition structure, then this predicts that they would exhibit a decreased tendency to choose that same option. The best account of the behavioral data in this task was provided by a hybrid model in which model-based and model-free predictions were integrated during learning (unless subjects had to accomplish a cognitively demanding dual-task, in which case model-free control becomes rampant (Otto et al., 2013). However, across subjects, there was a wide spread in the degree of dependence on each system. Unexpectedly, ventral striatal fMRI signal, a region that normally correlates with model-free temporal difference prediction errors, was found to covary also with a temporal difference prediction error calculated on the basis of model-based predictions. The extent of this covariation for an individual subject was correlated with the extent to which that subject’s behavior was model based. One reason for a surprise at the presence of this signal is that the model-based system is not thought to use these prediction errors for its own calculations (rather, it uses the state prediction error, where a state prediction error is a measure of the surprise in a new state given a current estimate of state-action-state transition probabilities (Gläscher et al., 2010). One suggested possibility here is that the model-based system is training the model-free system.

Along with these human studies, there is now an accumulating wealth of reports of the sort of neural response profile that would be predicted if indeed an animal is evaluating a menu of internally represented actions and their consequences at critical decision points. This is particularly true in spatial tasks (Johnson and Redish, 2007; Pfeiffer and Foster, 2013; van der Meer and Redish, 2009) and is a potential neural associate of the VTE behavior we mentioned above. In particular, at decision points such as a branch point in a maze, hippocampal place cell responses can be observed to sweep forward from the actual location of the subject. They do so in a manner consistent with the idea that the subject is engaged in some form of deliberation regarding its future potential states and the worth thereof (Johnson and Redish, 2007; Pfeiffer and Foster, 2013; van der Meer and Redish, 2009), for instance, being correlated with the subject’s ultimate choices. In a similar vein, a recent mouse study has reported that units in ventral hippocampus, a region which is strongly connected to those supporting reward processing, mediates a form of goal-oriented search (Ruediger et al., 2012).

The forward sweeps relevant to immediate choices are assumed to start at the subject’s current location. However, when an animal is not running in its environment, or indeed when it is sleeping, it is also possible to observe a variety of forward and backward sweeps (Dragoi and Buzsáki, 2006; Foster and Wilson, 2006, 2007; Lee and Wilson, 2002; Louie and Wilson, 2001) related to more or less recent experience in the world. It has been suggested that these are reflections of a model-based system training a model-free system, something that had been suggested in RL in the form of a technique called DYNA (Sutton, 1991). Backward sweeps (called reverse replay) seem particularly relevant for understanding the mechanisms supporting certain aspects of value learning, providing the means for the back propagation of value signals to the earliest predictor of their likely future occurrence, without needing a forward-looking prediction error (Foster and Wilson, 2006). One computational formulation that addresses this question is DYNA-Q (Sutton, 1990), which allows an agent to exploit previously recorded experience to update values and policies, an idea that has now been exploited in modeling studies (Johnson and Redish, 2005). One could argue that a decision-making counterpart of consolidation (which is a normal view of hippocampal replay; McClelland et al., 1995) is exactly a model-free instantiation of a policy.

### Generation 4: Elaborations on Model-Based and Model-free Control

With these prior generations as the foundation, a current set of studies is focusing on unearthing more about the interaction between model-based and model-free control (Doll et al., 2012) and indeed more about model-based control itself, given its manifest computational complexities. This is given added urgency by recent evidence that even the simplest type of instrumental learning task has model-based and model-free components (Collins and Frank, 2012).

First, there has been anatomical and pharmacological insight into the balance of influence between the two systems. For example, the strength of white matter connections between premotor cortex and posterior putamen is reported to predict vulnerability to “slips of action” (where non-goal-relevant, previously trained, actions are automatically elicited by environmental cues), a vulnerability also predicted by gray matter density in the putamen (de Wit et al., 2012b). Such slips have been considered as intrusions of habits. This contrasts with tract strength between caudate and ventromedial prefrontal cortex that predicted a disposition to express more flexible goal-directed action, evident in an ability to selectively respond to still rewarding outcomes (de Wit et al., 2012b).

Most work on the pharmacology of the different forms of control has centered on the neuromodulator dopamine. However, complexities are to be expected since dopamine is likely to play a role in both systems (Cools, 2011). First, as noted, the phasic firing of dopamine neurons has been suggested as reporting the temporal difference prediction error for reward (Montague et al., 1996; Schultz et al., 1997) that underpins model-free evaluation and control via its influence over activity and plasticity (Reynolds et al., 2001; Frank, 2005). Second, dopamine projects to the entire striatum, including regions such as dorsomedial striatum (or caudate), which have been implicated in model-based control, and dorsolateral striatum (or putamen), implicated in model-free control (Balleine, 2005). Indeed, lesions to nigrostriatal dopamine impair habit (stimulus-response) learning (Faure et al., 2005). Substantial work in conditions such as Parkinson’s disease, in which dopamine is reduced, shows that manipulations favoring D1 and D2 dopamine receptors result in effects that are most readily interpretable in a model-free manner (Frank et al., 2004). Third, dopamine exerts a significant influence over prefrontal cortical functions such as working memory (Williams and Goldman-Rakic, 1995), in a manner that depends on initial levels or efficacy of this neuromodulator (Cools and D’Esposito, 2011). These functions are particularly critical for the operation of model-based control. For instance, in a rat experiment in which a posttraining manipulation of value was coupled to a dopamine infusion into ventromedial PFC (vmPFC) (Hitchcott et al., 2007), a bidirectional effect was evident whereby the dopamine infusion decreased responding to a devalued outcome and enhanced responding to nondevalued outcomes, suggesting an influence on model-based valuation.

At a mechanistic level, dopamine is likely to affect model-based control via its impact on maintenance processes associated with the prefrontal cortex. For example, disrupting prefrontal function using TMS renders behavior more habitual (Smittenaar et al., 2013), while boosting dopaminergic function enhances psychological and electrophysiological signatures of such maintenance processes (Moran et al., 2011). This is consistent with the effects of dopamine on working memory in macaques (Williams and Goldman-Rakic, 1995) and also with the fact that manipulations of dopamine in prefrontal regions directly affect model-based control (Hitchcott et al., 2007). However, the extensive dopamine innervation of regions of the striatum devoted to goal-directed control suggests the possibility that control over working memory might not be its sole mode of influence (Frank et al., 2001).

Finally, in a modern experiment into the irrelevant incentive effect (Krieckhaus and Wolf, 1968), it was observed that sudden revaluation in Pavlovian conditioning is associated with dramatic upregulation of activity in dopaminergic nuclei as inferred from elevated Fos activity (along with many other regions, including the orbitofrontal cortex) (Robinson and Berridge, 2013). Specifically, rats who had learned repulsion to an unpleasant salt stimulus, when first reencountering this stimulus in a salt-deprived state, showed immediate attraction to this same stimulus. If one interprets revaluation in this context as depending on some form of model-based prediction (albeit not necessarily the same as instrumental model-based prediction; P.D. and K. Berridge, unpublished data), then this places dopamine at the heart also of the model-based system.

One indirect method to address the role played by dopamine in instrumental control in humans exploits a dopamine depletion technique, involving acute dietary phenylalanine and tyrosine depletion (APTD). de Wit et al. (2012a) used this manipulation in subjects performing a reward learning paradigm, employing outcome devaluation and measuring slips of action to assess the degree of model-based versus model-free control. After devaluation, depletion had no impact upon stimulus-response learning or response-outcome learning. Instead, depletion tipped the balance of control toward more habitual responding as revealed in a greater frequency of slips of action. However, depletion studies, whether experimental or disease based, are likely to exert a much less detrimental effect on dopamine function than the 6-OHDA lesions conventionally used in animal studies.

The frequency of slips of action does not offer a very precise measurement of the relative influence of model-based and model-free systems. In a double-blind, fully counterbalanced (repeated-measures), design, Wunderlich et al. (2012b) administered either L-DOPA (to boost the influence of dopamine) or placebo while subjects solved the two-step Markov decision task of (Daw et al., 2011). By fitting the same class of model as in the original study, the authors showed that subjects were more model based in their behavior when under L-DOPA, favoring the notion that the dominant influence of this type of dopaminergic manipulation is over prefrontal function rather than over dorsolateral striatal habits (Wunderlich et al., 2012b).

Conversely, Parkinson’s disease involves the progressive death of dopamine cells and so causes a decrease in dopamine release. de Wit and colleagues tested Parkinson’s patients in an instrumental conflict task in which response-outcome links associated with a model-based system would putatively impair performance in a critical set of (incongruent) trials, whereas model-free, stimulus-response, associations would be helpful (de Wit et al., 2011). They showed that subjects with the disease could solve the task, arguing that habit formation may not have been eliminated. They also showed that (goal-directed) performance in a posttraining devaluation test covaried negatively with disease severity, arguing that model-based influences were impaired. These results are consistent with the findings above, albeit harder to integrate with other notions about deficits in model-free learning in Parkinson’s patients.

Various new tasks have also shed light on model-based and model-free systems (Doll et al., 2012). For instance, Wunderlich and colleagues exposed subjects to a task with elements explicitly designed to engage each system (Wunderlich et al., 2012a). Here, in the element directed at model-free control, subjects were overtrained to make choices within four sets of pairs of options, based on experience of the probabilistic reward to which the options led. In the element directed at model-based control, they had to navigate a branching, three-step decision tree to reach one of several possible terminal states, each associated with an instructed probability of reward that changed on a trial-by-trial basis. Critically, the choice at the middle step was made by the computer playing a minimax strategy to ensure that subjects engaged in a form of model-based dynamic programming that involved estimating the values of distinct stages in the decision tree. Finally, while being scanned, subjects were faced with three different tasks: the full three-step decision tree; a choice between two overtrained pairs; or a choice between one overtrained pair and half a decision tree.

In the full three-step decision tree, anterior caudate nucleus BOLD covaried with optimal and alternative values, such that during a root branch decision, caudate activity related to several values relevant to the choice, including those present at consecutive choices deeper in the tree. However, during the third, deepest, choice, caudate activity was still associated with the values of both current choice alternatives but no longer with the value of the previously rejected root branch (Wunderlich et al., 2012a). This is exactly the pattern expected in a forward tree search during goal-directed (model-based) decision making, where values related to distinct options are prospectively represented. Notably, these model-based effects were not evident in another basal ganglia structure, the putamen, which only encoded model-free values for extensively trained options at the time of choice. By contrast, when subjects were required to choose between an overtrained pair and half the tree, a situation requiring access to both model-based and model-free values, the caudate represented the planned target value of the decision tree, while activity in the putamen pertained solely to the value of the overtrained pair.

This dissociation corresponds exactly to the response patterns of a model-free controller that depends on cached values (putamen) and a model-based controller that depends on values calculated on the fly (caudate). Thus, when goal-directed and habit-based options compete, the activity in caudate and putamen covaried with planned and cached values even under situations where the relevant actions were not chosen. The findings fit snugly with an animal literature both in terms of anatomical dissociations as well as findings that highlight both systems act synergistically and in parallel (Wassum et al., 2009). In stark contrast, activity in vmPFC encoded the winning outcome of the choice process (chosen value), irrespective of whether this choice was based on a model-based or model-free value. Thus, vmPFC can access both model-based and model-free values, consistent with parallel, and independent, operation of model-based and model-free valuation systems.

Simon and Daw designed a different, spatial, task in order to examine model-based inference (Simon and Daw, 2011). Here, subjects navigated a maze consisting of a set of rooms connected by one-way doors in order to get to goals; however, the structure of the maze changed randomly at every step, with the doors changing their allowed directions according to a small, fixed, probability. The constant change in the structure of the maze invited subjects to use model-based planning, and indeed their behavior was better fit by a model-based rather than a model-free method. Having pinned the behavior down, the authors were then in a position to study the neural representations of value signals associated with the planning task as well as other model-based quantities, such as the number of choices at the current and the next step in the maze (Simon and Daw, 2011). Regions such as ventrolateral and ventromedial putamen, whose BOLD signals are traditionally supposed to covary with model-free values or prediction errors, turned out to covary with key model-based value signal. By contrast, there was little evidence that BOLD in the vmPFC was also related to value, as might have been expected, though parts of the medial temporal lobe also showed significant correlations with these and with reward predictions. The authors suggested that the latter findings might possibly reflect the spatial nature of the task, compared with the more abstract Markov decision problems that had previously implicated the vmPFC. Other regions including the anterior insula, the precentral cortex, and the medial cingulate covaried with facets of the transitions available from a room, suggesting that they might be involved in realizing the model of the world. Tasks such as this have a strong spatial component, as opposed to the more abstract structure of many planning tasks, and this attribute might account for the presence of model-based signals seen in hippocampus and medial temporal lobe. In fact, this has a bearing on a suggestion that there is another form of controller, an “episodic controller,” that involves these very structures (Lengyel and Dayan, 2008).

Other illuminating paradigms include a so-called grid-sailing task (Fermin et al., 2010), which uses structurally different rules (in the form of key mappings) in a motor-learning task. This task has provided evidence that subjects use a model-based strategy to generalize learning. There is also a suggestion that explicit instructions and advice (whose immediate impact must surely be more model-based than model-free) operate by boosting the impact of model-free learning on trials on which instructions are followed (Doll et al., 2009). Also, of note is a recent implementation of an ingenious behavioral design, involving a simple one- and two-step problem in which learning and performance occurred in distinct phases (Gershman et al., 2012). In the final phase of the task, model-based and model-free controllers would make the same choices, albeit for different reasons. In fact, the authors observed that subjects acted in a manner consistent with a model-based system having trained by a model-free one during an earlier phase of learning, as in an online or offline form of the DYNA-Q algorithms mentioned above (Sutton, 1991). In effect, these findings highlight cooperation, as opposed to competition, between the two systems.

### Generation 5: The Future

There are many outstanding questions related to model-based and model-free control, and these are now the focus of intense investigation. In the remainder of this Review, we touch on some of the main strands of this research and the plethora of unresolved issues. These include how model-based control is realized; how the competition between model-based and model-free control is resolved when they disagree; the relationship between the formulation of model-based control that we have adopted and the many other related dichotomies; the relevant interactions between instrumental conditioning, which has so far been our central focus, and Pavlovian conditioning; and finally some early work on psychopathological interactions.

#### Model-Based Realization

The first critical issue is how model-based calculations are realized. Building and searching a deep tree imposes a huge burden on cognitive control and working memory. However, there is presently not much work that extends from hippocampal preplay in spatial domains (Johnson and Redish, 2007; Pfeiffer and Foster, 2013) to planning in multistep tasks (Wunderlich et al., 2012a; Simon and Daw, 2011). Nevertheless, the latter studies delivered neural evidence for tree-like calculations. Other related search tasks have found behavioral evidence for these calculations and have started to look at heuristics for pruning the tree, a necessity when it gets too wide or deep (Huys et al., 2012).

One general notion is to treat the problem of model-based evaluation as an internal decision problem (Dayan, 2012) with actions such as gating information into working memory (O’Reilly and Frank, 2006) or expanding a state in the tree in terms of the actions that are possible. These could depend sensitively on the hierarchical architectures of cognitive control in lateral and medial prefrontal regions and their striatal connections (Frank and Badre, 2012; Koechlin and Hyafil, 2007; Koechlin et al., 2003).

Adaptations of RL architectures such as DYNA-2 (Silver et al., 2008) may allow model-free values to be integrated with model-based values to circumvent the complexity of very deep trees (Sutton and Barto, 1998; Pezzulo et al., 2013); they might also provide a rationale for the observation that regions that are normally considered to report model-free temporal difference prediction errors can be invaded by prediction errors evaluated on the basis of model-based predictions (Daw et al., 2011). An alternative idea is to transform control-theoretic calculations of the optimal policy into the sort of probabilistic inference problems that are generally believed to be solved by sensory processing regions of the cortex in order to interpret input (Solway and Botvinick, 2012). The consilience is attractive; however, the calculational complexities largely remain (Pezzulo et al., 2013).

#### Competition between Model-Based and Model-free Control

In variants of an architecture such as DYNA-2 (Silver et al., 2008), there can be a seamless integration of model-based and model-free values of actions as part of the way that the former are calculated. Alternatively, if the model-based system mainly influences the model-free system by regurgitating examples (Dragoi and Buzsáki, 2006; Foster and Wilson, 2006, 2007) or selectively boosting its learning rate (Biele et al., 2011; Doll et al., 2009, 2011), and in so doing trains short-term model-free values, then the MF system could do its bidding and may not actually need explicitly to seize control. Otherwise one has to invoke some form of competitive combination of model-based and model-free values. Daw et al.’s finding that different subjects employ each system to a greater or lesser degree (Daw et al., 2011) might be seen as being evidence for the latter idea.

Various suggestions have been made for how arbitration should proceed, but this is an area where much more work is necessary. One idea is that it should depend on the relative uncertainties of the systems, trading the noise induced by the calculational difficulties of model-based control off against the noise induced by the sloth of learning of model-free control (Daw et al., 2005). This provides a natural account of the emergence of habitual behavior (Dickinson, 1985), as in the latter noise decreases as knowledge accumulates. By this account, it could be the continual uncertainty induced by the changing mazes in Simon and Daw (2011) that led to the persistent dominance of model-based control. Equally, the uncertainty associated with unforeseen circumstances might lead to the renewed dominance of model-based control, even after model-free control had asserted itself (Isoda and Hikosaka, 2011).

A different idea suggested by Keramati et al. (2011) starts from the observation that model-free values are fast to compute but potentially inaccurate, whereas model-based ones are slow to compute but typically more accurate (Keramati et al., 2011). They consider a regime in which the model-based values are essentially perfect and then perform a cost/benefit analysis to assess whether the value of this perfect information is sufficient to make it worth acquiring expensively. The model-free controller’s uncertainty about the relative values of the action becomes a measure of the potential benefit; and the opportunity cost of the time of calculation (quantified by the prevailing average reward rate (Niv et al., 2007) is a measure of the cost. A related suggestion involves integration of model-free and model-based values rather than selection and a different method of model-based calculation (Pezzulo et al., 2013).

#### Other Model-free and Model-Based Formulations

There is no unique form of model-free or model-based control and evidence hints that there are intermediate points on the spectrum between them. For instance, there are important differences between model-free control based on the predicted long-run values of actions (as in Q-learning) (Watkins, 1989), or SARSA (Rummery and Niranjan, 1994), and actor-critic control (Barto et al., 1983). In the latter, for which there is some interesting evidence (Li and Daw, 2011), action choice is based on propensities that convey strictly less information than the long-run values of those actions. There are even ideas that the spiraling connections between the striatum and the dopamine system (Joel and Weiner, 2000; Haber et al., 2000) could allow different forms of controller to be represented in different regions (Haruno and Kawato, 2006).

Intermediate points between model-based and model-free control can arise from temporally sophisticated representations of states that contain predictions about likely future states (thus being partly model based) but that can be used in a straightforward manner by the model-free controller, thereby including some facets of model-based control. One example is the successor representation (Dayan, 1993). Further, there are suggestions that there are multiple model-based controllers, i.e., a mixture model (Doya et al., 2002), in which the selection between them can have model-based or potentially model-free components.

Finally, there is a rich panoply of other formulations of the dichotomies between model-free and model-based control and of model-based control itself (Dayan, 2009; Kahneman, 2011; Stanovich and West, 2002). We have already seen some variants, with the issue of instruction versus experience (as in Wunderlich et al., 2012a) but there are many others too, including declarative versus procedural, spatial/geometric versus abstract, interpreted versus compiled, prior- versus data-bound (Dayan, 2009), and even episodic versus semantic control (Lengyel and Dayan, 2008). Teasing these various aspects apart, and understanding what properties and substrates they share, is critical. For example, iterations of reflective control as captured by ideas such as model based, declarative, and goal directed are almost certainly not fully commensurable.

#### Pavlovian Conditioning

So far, we have concentrated on instrumental control, i.e., the choice of actions based on their past or current contingencies. Another, even more influential source of control is Pavlovian, in which predictions of future valenced outcomes lead automatically to a choice of action (such as approach for appetitive outcomes and inhibition or withdrawal for aversive ones) irrespective of the benefit of that action (Dayan et al., 2006; Williams and Williams, 1969). One way to conceive of these Pavlovian systems is in terms of an evolutionarily specified prior, serving to facilitate performance by alleviating the computational costs that come with instrumental conditioning’s increased flexibility in being able to learn to emit arbitrary actions.

There is good evidence for Pavlovian predictions of actual outcomes, which what we argue underpins instrumental model-based control, and this seems to account for behavioral phenomena such as specific forms of Pavlovian instrumental transfer (PIT) (Ostlund and Maidment, 2012; Kruse et al., 1983). However, there are two key additional aspects to Pavlovian conditioning. First is the idea that Pavlovian control might influence instrumental model-based calculations. For instance, we noted above that building and evaluating the tree might be considered in terms of a set of internal actions (Dayan, 2012). Those actions might also be susceptible to Pavlovian biases. One example is the possibility that pruning of the decision tree, which we argued is likely to be of great importance in the face of its size, might be subject to Pavlovian manipulation. It could, for instance, happen automatically in the face of potential punishments, even when this pruning is suboptimal (Huys et al., 2012).

Second, Pavlovian conditioning differs from instrumental conditioning conceptually in the choice of action (automatic versus learned) rather than in the nature of the predictions, and so it is possible that it also has access to both model-free and model-based predictions. This is important for interpreting a range of Pavlovian conditioning results, such as the difference between identity unblocking, which is outcome specific (McDannald et al., 2011) and so putatively model based, versus valence unblocking, which is outcome general and so model free.

As a final example, consider Pavlovian to instrumental transfer (PIT), in which Pavlovian cues modify the vigor of instrumental responding as, for example, when appetitive cues increase responding for reward. PIT comes in two flavors: specific and general. Specific PIT depends on a match between the particular outcome that is expected as both the Pavlovian and instrumental target and so appears to be model based. Conversely, general PIT depends solely on the valence of the Pavlovian cue, as expected for a model-free prediction. This distinction has been used to good effect in determining the substrates of model-based and model-free predictions (Balleine, 2005), for instance, differentiating the role of basolateral and central nuclei of the amygdala and their connections to the core and shell of the nucleus accumbens.

Many early fMRI studies into prediction errors used model-free accounts in Pavlovian paradigms and located prediction errors in striatal BOLD (Berns et al., 2001; O’Doherty, 2004; O’Doherty et al., 2003; Haruno et al., 2004). More recent investigations have looked closely at the distinction between model-based and model-free, detecting evidence for the former in areas such as the amygdala (Prévost et al., 2013). However, it is not clear that Pavlovian and instrumental model-based predictions are the same (P.D. and K. Berridge, unpublished data). For instance, instant Pavlovian revaluation associated with saline deprivation happens normally in decorticate animals, evidently not depending on regions strongly affiliated with model-based control such as the vmPFC (Wirsig and Grill, 1982). Further, there are dissociations between the effect of devaluation in instrumental responding versus PIT (Holland, 2004), and the irrelevant incentive effect, which shows a form of model-based motivationally sensitive evaluation, appears to depend on something akin to PIT (Dickinson and Dawson, 1987a, 1987b) in a way that suggests this Pavlovian/instrumental difference.

#### Psychopathology

How control is parsed between model-based and model-free systems is likely to have psychopathological implications. There is currently great interest in using the sorts of ideas and tasks that we have discussed to provide a quantitative way of understanding the nature and underpinnings of abnormal decisions, choices, and evaluations. The suggestion that systems occupy something closer to a spectrum than a dichotomy makes this a potentially powerful way to parse deviance but also very challenging.

One example is obsessive-compulsive disorder (OCD) (Graybiel, 2008), where insensitivity to outcome devaluation and slips of action were used to test a hypothesis of dominance by a habitual system (Gillan et al., 2011). Patients with OCD (albeit potentially confounded by the effects of their neuromodulatory therapies) showed no deficit in using rewarding feedback to guide action but instead showed both lack of sensitivity to outcome devaluation and increased frequency in slips of action. A similar conclusion has been derived from observations of the two-step task (Daw et al., 2011) in OCD patients, as they, along with substance abusers and binge eaters, showed a lower dependence on model-based control (V. Voon, personal communication). Furthermore, evidence for abnormalities in components of a goal-directed system in OCD, particularly the caudate nucleus, aligns with a suggestion that key manifestations of this condition reflect on overdominance of a habitual system (Maia et al., 2008).

A second example is drug addiction (Belin et al., 2009). One influential proposal is that a protracted exposure to addictive drugs recruits dopamine-dependent striato-nigro-striatal ascending spirals (Haber et al., 2000; Joel and Weiner, 2000) from the nucleus accumbens to more dorsal regions of the striatum (Everitt et al., 2008). This results in a shift in control from action-outcome to stimulus-response mechanisms, a putative dominant mode of control in drug seeking and drug relapse. What this entails is that a key mechanism underlying the emergence of compulsive drug seeking, as well as relapse into addictive behaviors, is the subversion of control by a contextually dominant habitual mode.

A final question here relates to the consequence of overdominance of a model-based system. Speculatively, we suggest that it might at least be involved in components of the phenomenology seen in psychotic states, such as paranoia, delusions, and hallucinations. The latter can be seen as arising when the sort of processes that are associated with building and evaluating a model become sufficiently detached from external input from the world. We observed that boosting dopamine boosts the impact and control of such model-based influences (Wunderlich et al., 2012b) and perhaps this is at least one pathophysiological step. It is worth noting that in the treatment of Parkinson’s disease, boosting dopamine function often leads to the emergence of psychotic phenomena (Yaryura-Tobias et al., 1970).

### Conclusion

We have provided an inevitably selective Review of the past, present, and future of model-based and model-free control in humans. The distinction is extremely long standing, has been an important source of ideas and experiments, has offered accounts of many brain regions critical to instrumental choice, and indeed has been a spur to computational modeling. However, even though it is not yet evident how the computational challenges of model-based control are addressed, it is becoming clear that model-based and model-free predictions and controls are more richly intertwined than originally supposed and thereby offer flexible and adaptive solutions to the manifest problems of exploring and exploiting potentially dangerous but lucrative environments.
